# Autophagy Protects against CYP2E1/Chronic Ethanol-Induced Hepatotoxicity

**DOI:** 10.3390/biom5042659

**Published:** 2015-10-16

**Authors:** Yongke Lu, Arthur I. Cederbaum

**Affiliations:** 1 Department of Structural and Chemical Biology, Icahn School of Medicine at Mount Sinai, New York, NY 10029, USA; E-Mail: luyongke@yahoo.com; 2 Department of Pharmacology and Systems Therapeutics, Icahn School of Medicine at Mount Sinai, New York, NY 10029 , USA

**Keywords:** chronic ethanol, cytochrome P450 2E1 (CYP2E1), autophagy protection, hepatotoxicity, oxidative stress, 3-methyladenine, rapamycin

## Abstract

Autophagy is an intracellular pathway by which lysosomes degrade and recycle long-lived proteins and cellular organelles. The effects of ethanol on autophagy are complex but recent studies have shown that autophagy serves a protective function against ethanol-induced liver injury. Autophagy was found to also be protective against CYP2E1-dependent toxicity *in vitro* in HepG2 cells which express CYP2E1 and *in vivo* in an acute alcohol/CYPE1-dependent liver injury model. The goal of the current report was to extend the previous *in vitro* and acute *in vivo* experiments to a chronic ethanol model to evaluate whether autophagy is also protective against CYP2E1-dependent liver injury in a chronic ethanol-fed mouse model. Wild type (WT), CYP2E1 knockout (KO) or CYP2E1 humanized transgenic knockin (KI), mice were fed an ethanol liquid diet or control dextrose diet for four weeks. In the last week, some mice received either saline or 3-methyladenine (3-MA), an inhibitor of autophagy, or rapamycin, which stimulates autophagy. Inhibition of autophagy by 3-MA potentiated the ethanol-induced increases in serum transaminase and triglyceride levels in the WT and KI mice but not KO mice, while rapamycin prevented the ethanol liver injury. Treatment with 3-MA enhanced the ethanol-induced fat accumulation in WT mice and caused necrosis in the KI mice; little or no effect was found in the ethanol-fed KO mice or any of the dextrose-fed mice. 3-MA treatment further lowered the ethanol-decrease in hepatic GSH levels and further increased formation of TBARS in WT and KI mice, whereas rapamycin blunted these effects of ethanol. Neither 3-MA nor rapamycin treatment affected CYP2E1 catalytic activity or content or the induction CYP2E1 by ethanol. The 3-MA treatment decreased levels of Beclin-1 and Atg 7 but increased levels of p62 in the ethanol-fed WT and KI mice whereas rapamycin had the opposite effects, validating inhibition and stimulation of autophagy, respectively. These results suggest that autophagy is protective against CYP2E1-dependent liver injury in a chronic ethanol-fed mouse model. We speculate that autophagy-dependent processes such as mitophagy and lipophagy help to minimize ethanol-induced CYP2E1-dependent oxidative stress and therefore the subsequent liver injury and steatosis. Attempts to stimulate autophagy may be helpful in lowering ethanol and CYP2E1-dependent liver toxicity.

## 1. Introduction

Autophagy is an intracellular pathway by which lysosomes degrade and recycle long-lived proteins and cellular organelles. This pathway degrades cellular components that are damaged or are needed to generate substrates that maintain cellular energy homeostasis under conditions of limited nutrients or stress [1,2,3,4,5]. The regulation of this process is complex and controlled by the coordinated actions of autophagy-related genes (Atgs). Removal of damaged mitochondria by mitophagy or of lipid droplets by lipophagy are selective forms of macroautophagy [6,7,8]. Removal of damaged mitochondria protects the cell against mitochondrial oxidative stress, while removal of lipid droplets limits the accumulation of lipids by hepatocytes. Defects in lipophagy can contribute to hepatic steatosis. Interestingly, hepatocyte lipid accumulation also decreases autophagy, indicating that steatosis might be a mechanism of defective hepatic autophagy [6,9,10]. Autophagy is decreased in certain hepatic and pancreatic diseases e.g., α1-antitrypsin deficiency and non-alcoholic fatty liver disease, but is increased in nutrient deficiency and hepatitis B infection [8,10,11]. In general, autophagy is considered as a cell survival pathway but one that can also mediate cell death under certain conditions or when overactivated.

Alcohol-induced liver injury (ALD) is a significant global health problem and a leading cause of death worldwide. The mechanisms by which ethanol treatment causes cell death are still not clear. Many of the hepatic toxic effects of ethanol have been linked to its metabolism in the liver. Ethanol-induced liver pathology has been shown to correlate with CYP2E1 levels and lipid peroxidation [12,13,14,15,16]. The biochemical and toxicological properties of CYP2E1 have been widely studied [17,18,19,20]. CYP2E1 is induced under a variety of pathophysiological conditions such as fasting, diabetes, obesity and high fat diet, by drugs, in non-alcohol-induced steatohepatitis and by alcohol [21,22,23,24,25,26]. Autophagy can either be increased or decreased by ethanol depending on the model used, the dose, the tissue evaluated and the experimental condition [27,28,29,30,31,32,33,34,35]. While the effects of ethanol on autophagy are complex and require further study, it is becoming clear that autophagy serves a protective function against ethanol-induced liver injury [4,28,29]. Chronic ethanol consumption was recently shown to increase autophagy [31,34] and in some cases this increase was associated with a decrease in activity of the other major cellular proteolytic system, the proteasome complex [35].

To our knowledge, there have been no reports as to whether macroautophagy is protective against CYP2E1 elevation of ROS, fatty liver and liver injury after chronic ethanol treatment or promotes these responses by the liver to ethanol. The rationale for this is that CYP2E1 plays a role in ethanol-induced oxidant stress, fatty liver and liver injury. Autophagy in some settings is protective against cell injury, while in other settings, autophagy can promote cell toxicity. If autophagy is protective against chronic ethanol/CYP2E1 toxicity, attempts to stimulate autophagy may prove to be helpful in lowering ethanol-induced liver injury. If autophagy promotes ethanol/CYP2E1 toxicity, inhibitors of autophagy may help to ameliorate ethanol hepatotoxicity.

We have recently shown that ethanol-induced toxicity, fat accumulation and generation of reactive oxygen species in HepG2 E47 cells which express CYP2E1 was potentiated by 3-methyladenine, an inhibitor of autophagy but decreased by rapamycin, which stimulates autophagy [36]. No effect was found with C34 control HepG2 cells which do not express CYP2E1. Similarly, the toxicity of several agents activated by CYP2E1 such as CCL_4_ or a polyunsaturated fatty acid such as arachidonic acid or depletion of GSH by buthionine sulfoximine in the E47 cells was enhanced by 3-MA or by SiRNA against Atg 7 [37]. In an acute binge alcohol model, there was modest liver injury, fat accumulation and increased ROS production in wild type and CYP2E1 knockin mice but not in CYP2E1 knockout mice [38]. These effects were magnified when 3-MA was also administered with the acute ethanol but were blunted when rapamycin was given with the ethanol [38]. These experiments are supportive of autophagy as being protective against ethanol/CYP2E1-dependent liver toxicity. The goal of the current report was to extend the previous *in vitro* and acute *in vivo* experiments to a chronic ethanol model to evaluate whether autophagy is also protective against CYP2E1-dependent liver injury after chronic ethanol treatment.

## 2. Results and Discussion

### 2.1. Results

#### 2.1.1. Effect of Chronic Ethanol Treatment on Liver Injury in WT, KO and KI Mice

**Figure 1:**
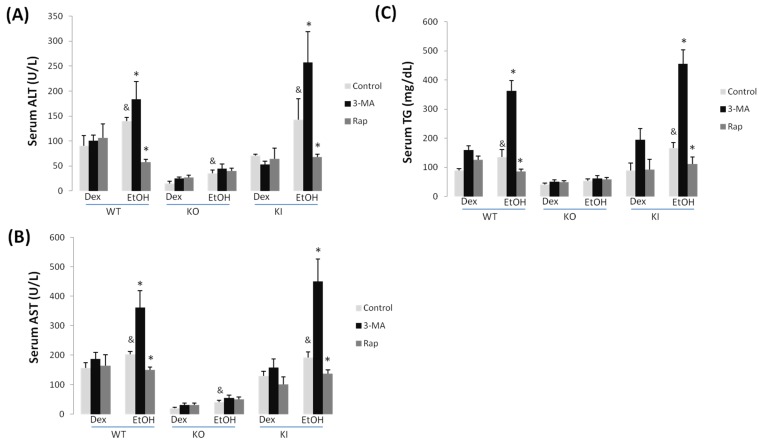
3-MA promotes but rapamycin prevents chronic ethanol induced liver injury in WT and KI mice. WT, KO and KI mice were fed with a Lieber-DeCarli liquid diet for four weeks. In the last week some mice were received an IP injection of saline, 3-MA or rapamycin once a day for seven days. ALT (**A**) and AST transaminase activities (**B**) and levels of triglyceride (TG) (**C**) were determined in the serum. &, *p* < 0.05 compared with Dextrose control, *n* = 4; *, *p* < 0.05 compared with alcohol alone-treated mice, *N* = 4.

Chronic ethanol feeding produced small increases in serum ALT and AST levels in all mice; highest levels (about 150–200 units/L) were found with the WT and KI mice as compared to the KO mice (30–40 units/L) (Figure 1A,B). Serum TG levels were significantly elevated by the chronic ethanol feeding to WT and KI mice but not to the KO mice (Figure 1C).

**Figure 2:**
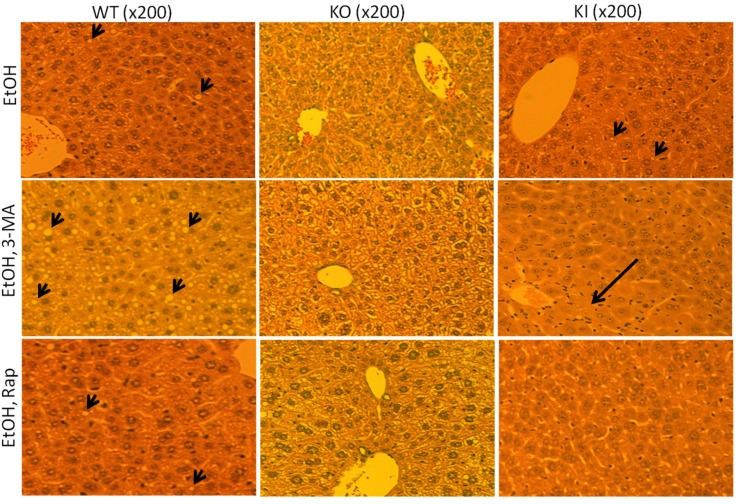
3-MA enhances liver toxicity in WT mice and KI mice whereas rapamycin is protective against liver injury. Chronic alcohol fed WT, KO and KI mice were treated with saline, 3-MA or rapamycin as mentioned in the legend to Figure 1. Liver slides were prepared for H&E staining and the extent of liver injury including steatosis and necrosis was observed under a light microscope. Arrowheads point to lipid droplets, and the arrow points to necroinflammation.

Histological evaluation showed no changes in liver pathology in any of the dextrose-fed mice [39]. The ethanol feeding produced an increase in liver fat accumulation in the WT and KI mice, but not the KO mice as compared to the dextrose-fed mice (Figure 2).

The steatosis scores were the following: WT-dextrose 0.95 ± 0.20; WT- ethanol 3.2 ± 0.6: KO-dextrose 0.5 ± 0.1; KO-ethanol 0.6 ± 0.1: KI-dextrose 0.2 ± 0.05; KI-ethanol 1.8 ± 0.4. The necroinflammation scores were 2 ± 0.6 for the ethanol-fed KI mice and below 0.2 for all the others. These results support the concept that CYP2E1 contributes to ethanol-induced liver injury and fat accumulation.

#### 2.1.2. Effect of 3-MA and Rapamycin on Chronic Ethanol-Induced Liver Injury

Treatment with 3-MA caused a significant further increase in ALT and AST levels in the ethanol-fed WT and KI mice but not the KO mice (Figure 1A,B). There was no effect by 3-MA in any of the dextrose-fed mice on serum transaminase levels. Treatment with rapamycin produced a decrease in ALT and AST levels in the ethanol-fed WT and KI mice but had no effect in the KO mice fed ethanol or any of the dextrose-fed mice (Figure 1A,B). The combined treatment with ethanol plus 3-MA elevated serum TG three-fold over the increase produced by ethanol alone in the WT and KI mice, but not the KO mice. In contrast, the combined treatment of ethanol plus rapamycin decreased serum TG levels in the WT and KI mice as compared to ethanol alone feeding; no effect by rapamycin was found in the ethanol-fed KO mice or in any of the dextrose-fed mice (Figure 1C).

The 3-MA treatment further elevated the steatosis in the ethanol-fed WT but not KO mice, and produced necrosis in the ethanol-fed KI mice (Figure 2). Treatment with rapamycin blunted the steatosis in the ethanol-fed WT mice and the necrosis in the ethanol-fed KI mice without any effect in the KO mice (Figure 2). No liver pathology was observed in any of the dextrose-fed mice either in the absence or presence of 3-MA or rapamycin.

#### 2.1.3. Effect of 3-MA and Rapamycin on Chronic Ethanol-Induced Oxidative Stress and CYP2E1

**Figure 3:**
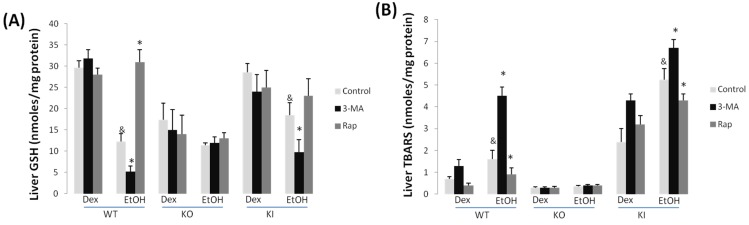
3-MA enhances while rapamycin blunts oxidative stress in chronic alcohol-fed WT and KI mice. GSH levels (**A**) in WT, KO and KI mice. 3-MA potentiates the decline in GSH produced by ethanol whereas rapamycin prevents this decrease of GSH in WT and KI mice; TBARS (**B**) are elevated by ethanol feeding in WT and KI but not in KO mice. 3-MA enhances ethanol induced TBARS but rapamycin lowers this increase. &, *p* < 0.05 compared with dextrose control; *, *p* < 0.05 compared with chronic alcohol alone, *N* = 4.

Chronic ethanol feeding produced a decrease in total GSH levels in the liver ranging from 65% in the WT mice to 30% in the KO mice (Figure 3A).

Treatment with 3-MA further lowered GSH levels in the ethanol-fed WT and KI mice but not the KO mice, whereas treatment with rapamycin blunted the ethanol-induced lowering of hepatic GSH levels (Figure 3A). Neither 3-MA nor rapamycin affected GSH levels in any of the dextrose-fed mice. Hepatic lipid peroxidation, assayed via formation of TBARS, was elevated two-fold by the chronic ethanol feeding to WT and to KI mice with no significant effect found with the KO mice (Figure 3B). The treatment with 3-MA potentiated the ethanol elevation of TBARS in the WT and KI mice but not the KO mice, whereas, in contrast, the treatment with rapamycin lowered the ethanol elevation of TBARS in these mice (Figure 3B). No effects by 3-MA or rapamycin were observed in any of the dextrose-fed mice.

**Figure 4:**
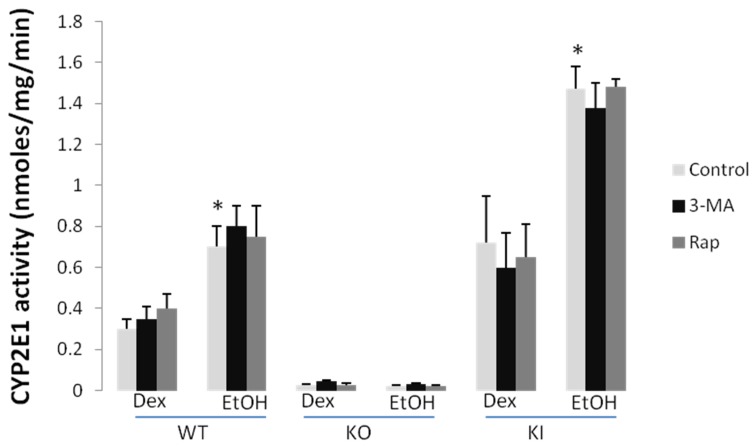
3-MA and rapamycin administration do not modify CYP2E1 catalytic activity. Liver microsomes were prepared from dextrose and ethanol-fed WT KO and KI mice treated with or without 3-MA or rapamycin treatment. CYP2E1 activity was determined using 4-nitrophenol (PNP) as the substrate. *, *p* < 0.05 compared with dextrose control, *N* = 3.

CYP2E1 catalytic activity was increased by the chronic ethanol feeding in the WT and KI mice; as expected, little or no activity was seen in the KO mice (Figure 4). Neither treatment with 3-MA nor with rapamycin has any effect on the basal CYP2E1 activity found in any of the dextrose-fed mice or in the elevated activity found with the ethanol-fed WT and KI mice (Figure 4). Immunoblots to assay the levels of CYP2E1 protein confirmed that neither 3-MA nor rapamycin had any effect on the levels of CYP2E1 in the dextrose-fed WT or KI mice or the ethanol-fed WT or KI mice [38].

#### 2.1.4. Effects of 3-MA and Rapamycin on Levels of Autophagic Proteins.

**Figure 5:**
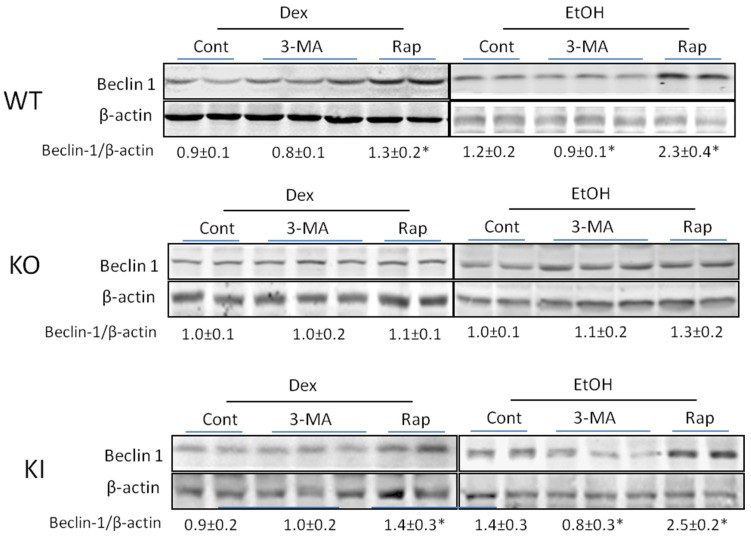
Effect of chronic ethanol or ethanol plus 3-MA or rapamycin on levels of Beclin-1. Beclin-1 levels in WT, KO and KI mice fed with alcohol or dextrose with or without 3-MA or rapamycin administration were determined by immunoblots. *, *p* < 0.05 compared with alcohol-alone fed or dextrose-alone fed mice. Results are calculated from four mice in each group, *N* = 4, although two to three blots are actually shown.

To validate that 3-MA or rapamycin were stimulating or inhibiting autophagy, respectively, in the chronic ethanol-fed mice and whether there was any modulation of their actions by CYP2E1, levels of key proteins involved in the autophagic process such as Beclin-1, Atg 7 or p62 were determined. As shown in Figure 5, 3-MA treatment caused a small but significant decline in the Beclin-1/β-actin ratio in the ethanol-fed WT and KI mice.

**Figure 6:**
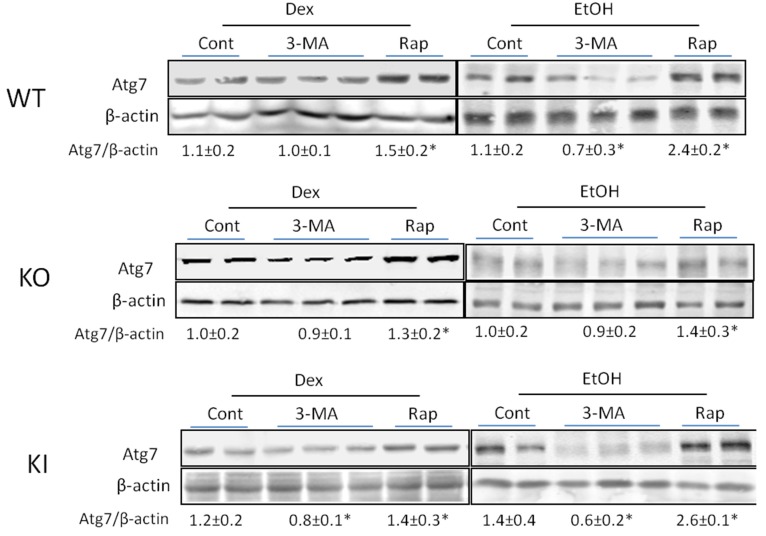
Effect of chronic ethanol or ethanol plus 3-MA or rapamycin on levels of Atg 7. Atg 7 levels in WT, KO and KI mice fed with alcohol or dextrose with or without 3-MA or rapamycin administration were determined by immunoblots. *, *p* < 0.05 compared with alcohol-fed alone or dextrose fed alone mice. Results are calculated from four mice in each group, *N* = 4, although two to three blots are shown.

Rapamycin produced an increase in Beclin-1 in the ethanol-fed WT and KI mice, and a small increase in the dextrose-fed WT and KI mice. No effects by 3-MA or rapamycin were found with the ethanol- or dextrose-fed KO mice. Similar results were found with respect to levels of Atg 7 as 3-MA produced a decrease in the Atg 7/β-actin ratio in the ethanol-fed WT and KI mice whereas rapamycin elevated this ratio more than two-fold in the WT and KI mice (Figure 6). 3-MA had no effect on Atg 7 levels in the ethanol or dextrose fed KO mice (Figure 6). There was a smaller but still significant increase by rapamycin in Atg 7 levels in the ethanol-fed KO mice (Figure 6) as well as very small increases in the dextrose-fed mice.

**Figure 7:**
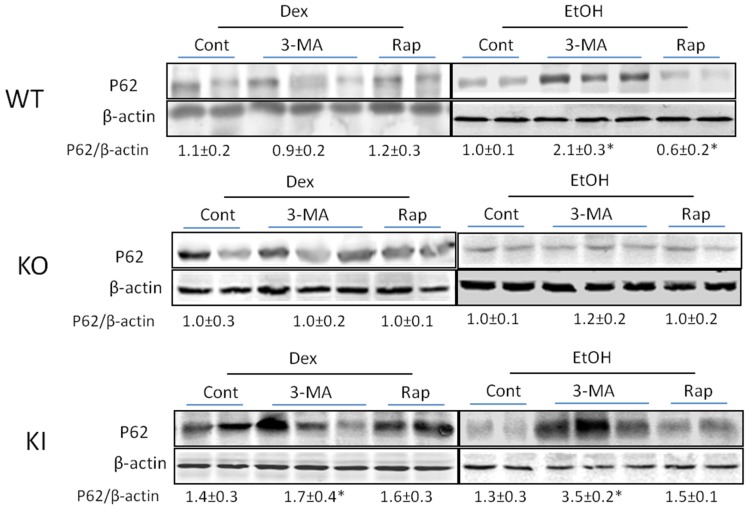
Effect of chronic ethanol or ethanol plus 3-MA or rapamycin on levels of p62. p62 levels in WT, KO and KI mice fed with alcohol or dextrose with or without 3-MA or rapamycin administration were evaluated by immunoblots. *, *p* < 0.05 compared with ethanol alone-fed mice.

With respect to levels of p62 which typically shows the opposite response as do Beclin-1 and Atg 7 autophagic proteins to modulators of autophagy, the 3-MA treatment increased the p62/β-actin ratio in the ethanol-fed WT and KI mice but not the KO mice (Figure 7). Rapamycin produced a decline in levels of p62 in the ethanol-fed WT mice but had no effect in the KI mice or the KO mice (Figure 7).

Taken as a whole, the decrease in Beclin-1 and Atg 7 coupled to the increase in p62 supports the inhibition of autophagy by 3-MA, while the opposite effects of rapamycin are supportive of a stimulatory effect on autophagy by this compound.

## 3. Discussion

Ding and colleagues [28,40] were among the first to report that autophagy protects against liver injury from acute alcohol *in vivo* and *in vitro* addition of ethanol to hepatocytes, and suggested that in response to alcohol, the liver might upregulate autophagy to selectively remove damaged mitochondria and to limit lipid accumulation. FOXO3a appears to play a critical role in this acute ethanol upregulation of autophagy [41]. Eid *et al.* [34] showed that autophagy was elevated by chronic ethanol feeding to rats and Yin and colleagues [31] reported that autophagic flux was increased by chronic ethanol feeding to mice for four weeks. The chronic ethanol-induced fat accumulation and liver injury was enhanced when autophagy was decreased by chloroquine but decreased when autophagy was increased by carbamazepine [31]. This protective action of autophagy was extended to nonalcoholic fatty liver [31]. Autophagy also was protective against acetaminophen toxicity [42], a drug which is metabolically activated by several cytochrome P450s, especially CYP2E1. Autophagy was protective against ethanol/CYP2E1-dependent toxicity in HepG2 cells *in vitro* and against an ethanol/CYP2E1 binge acute model in mice *in vivo* [36,37,38,43].

This protective action of autophagy against ethanol/CYP2E1 toxicity was extended in the current study to a chronic ethanol feeding model. Inhibition of autophagy by treatment with 3-MA increased the chronic ethanol elevation of serum transaminase and TG levels in WT and KI mice over the increases produced by ethanol alone. Stimulation of autophagy by treatment with rapamycin lowered the ethanol-induced elevation of serum transaminases and levels of TG in WT and KI mice. Smaller (ALT, AST) or little (TG) increase was produced by ethanol in the KO mice, and the treatments with 3-MA or rapamycin had little or no effect on these parameters in the KO mice. Histopathology showed fat accumulation in the ethanol-fed WT and necrosis in the ethanol-fed KI mice but not the KO mice. Fat accumulation was increased when autophagy was inhibited by 3-MA but decreased when autophagy was stimulated by rapamycin. The 3-MA treatment of ethanol-fed KI mice resulted in liver necrosis rather than more fat accumulation attesting to the protective effects of autophagy against chronic ethanol/CYP2E1 liver injury. Neither inhibition nor stimulation of autophagy by 3-MA or rapamycin had any effects on the basal CYP2E1 catalytic activity or protein levels in the dextrose-fed mice or on the increased catalytic activity and protein levels in the ethanol-fed mice. In this respect, the proteasome complex, not autophagy, has been shown to be the major protease system promoting degradation of CYP2E1 and ethanol induction of CYP2E1 is largely due to ethanol stabilization of CYP2E1 against proteasome-mediated turnover [44,45,46].

**Scheme 1:**
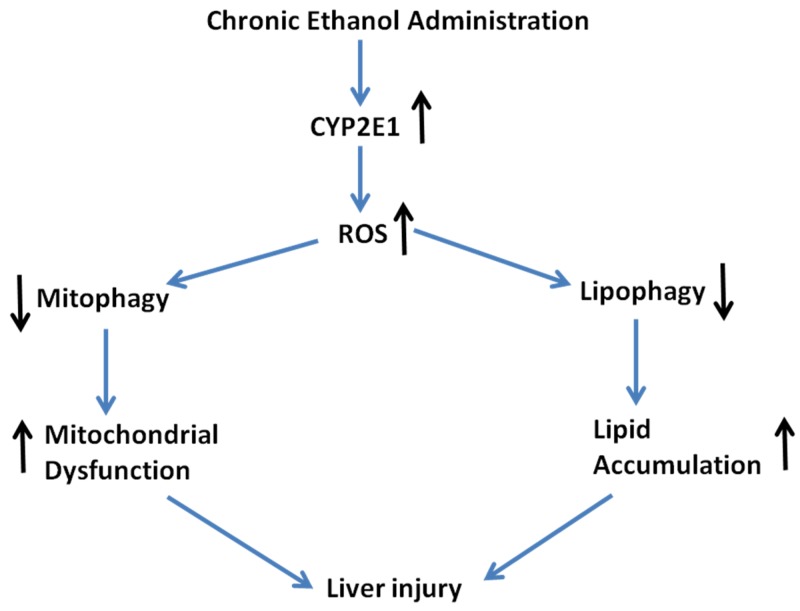
Model for chronic ethanol/CYP2E1-induced hepatotoxicity.

Autophagy may be activated by ROS as a cytoprotective mechanism but overactivation of autophagy by ROS could lead to cell injury [47,48,49,50]. However, not all ROS generators increase autophagy [51] and under some conditions ROS can inhibit autophagy [47,52,53]. CYP2E1 contributes to mechanisms by which alcohol produces oxidative stress [39,54,55]. Chronic ethanol feeding caused a decline in hepatic GSH levels and an increase in liver TBARS. Inhibition of autophagy by treatment with 3-MA caused a further decline in GSH levels in WT and KI mice while stimulation of autophagy with rapamycin prevented the decline in GSH and the increase in TBARs in WT and KI mice. Neither 3-MA nor rapamycin had any effect on levels of GSH or TBARs in the ethanol-fed KO mice. These results suggest that autophagy protects against chronic ethanol/CYP2E1-induced oxidative stress. Toxicity by arachidonic acid in HepG2 E47 cells was increased when autophagy was inhibited by SiRNA Atg 7 in association with an elevation in ROS [37]. The antioxidant *N*-acetyl cysteine decreased this toxicity and the elevated ROS and toxicity when autophagy was inhibited suggesting that ROS plays a role in the potentiation of CYP2E1 toxicity when autophagy is inhibited. Although mechanisms by which autophagy lowers the chronic ethanol/CYP2E1-induced oxidant stress were not evaluated in the current study, neither CYP2E1 catalytic activity or levels or the increase by ethanol were affected by 3-MA or rapamycin. We speculate that removal of damaged mitochondria by mitophagy may be one mechanism for the prevention of oxidative stress by autophagy. Studies on mitochondrial structure, function and bioenergetics will be helpful to assess this possibility. Removal of accumulated lipid droplets by lipophagy may contribute to the decrease in oxidant stress and levels of TBARS by autophagy. Autophagy-mediated lowering of oxidative stress is likely to play a key role in the protection against CYP2E1-dependent liver injury after chronic ethanol treatment. A simple scheme (Scheme 1) summarizing these results is shown below: The induction of CYP2E1 by ethanol elevated ROS which play a role in damage to mitochondria and to lipid accumulation. Removal of lipids and oxidized mitochondria by lipophagy and mitophagy prevents or lowers liver cell injury whereas blocking these processes potentiates cell injury.

Immunoblot analysis showed that 3-MA treatment lowered the levels of Beclin-1 and Atg 7 while increasing the levels of p62 in the chronic ethanol-fed mice reflective of inhibition of autophagy. Rapamycin increased the levels of Beclin-1 and Atg 7 in these mice suggesting stimulation of autophagy. Smaller or no effects by 3-MA or rapamycin were found with the KO mice in which the ethanol feeding itself had little effect.

We have previously reported that binge alcohol treatment of KI mice or acute alcohol treatment of WT mice lowers the LC3-II/LC3-I ratio and the levels of Beclin-1 and Atg 7 but increases the levels of p62 and mTOR as compared to saline controls [38]. Little or no effect on levels of these proteins was found in binge ethanol KO mice [38]. We interpreted these results as an inhibition of autophagic proteins by alcohol similar to conclusions of others who found that ethanol lowered LC3-II and Beclin-1 levels in hepatocytes and HepG2 cells [56] or in immune cells [30] or in a rat hepatoma cell line [32]. Such an interpretation is not consistent with reports that acute and chronic ethanol treatment increases autophagy and autophagic flux [28,31,34]. However, autophagic flux was not assayed in the studies reporting ethanol lowering autophagic protein levels, including our studies. Lowering levels of autophagic proteins could reflect a decline in autophagy but also could be due to increased autophagic degradation via elevated autophagic flux. Further studies will be necessary to assay for actual autophagic flux in ethanol-fed or acute ethanol-treated WT, KI and KO mice to evaluate whether CYP2E1 plays a role in the effects of ethanol on autophagy. Based upon the studies showing that acute [38] and chronic (this study) ethanol alters levels of Beclin-1 or Atg 7 or p62 in WT and KI mice to a greater extent than in KO mice, CYP2E1 or CYP2E1-derived ROS appears to play a modulatory role in the effects of ethanol on autophagy.

## 4. Experimental Section

### 4.1. Animal Model and Treatment

Wild type (WT) SV/129 mice were purchased from Charles River Laboratory. SV/129 background CYP2E1 knockout (KO) and humanized CYP2E1 transgenic mice (knockin, KI) mice [57,58] were a generous gift from Dr. Frank J. Gonzalez (Laboratory of Metabolism, National Cancer Institute, Bethesda, MD, USA) and colonies were established at Icahn School of Medicine at Mount Sinai. All mice were housed in temperature-controlled animal facilities with 12-h light/12-h dark cycles and were permitted consumption of tap water and Purina standard chow ad libitum until being fed the liquid diets. The mice received humane care, and experiments were carried out according to the criteria outlined in the Guide for the Care and Use of Laboratory Animals and with approval of the Mount Sinai Animal Care and use Committee.

Twenty-four male mice, body weight, 25–30 g, of each genotype were initially fed the control Lieber-DeCarli liquid dextrose diet (Bio-Serve, Flemingtown, NJ, USA) for 3 days to acclimate them to the liquid diet. Afterward, half of the mice of each genotype (*N* = 12) were fed the Lieber-DeCarli liquid ethanol diet (Bio-Serve) and the other half were fed the control liquid dextrose diet for a total of four weeks. The content of ethanol was gradually increased every 3 days from 10% (1.77% vol/vol) of total calories to 20% (3.54% vol/vol), 25% (4.42% vol/vol) and finally 30% (5.31% vol/vol). The control mice were pair-fed the dextrose diet on an isoenergetic basis. The ethanol-fed mice had access to their diets *ad libitum*, and the conditions of WT, KO, and KI mice were comparable. The amount of food consumed by WT, KO and KI mice was approximately the same. In the fourth week, each ethanol and dextrose diet fed group was divided into three subgroups. One group (*N* = 8) received saline ip, the second group (*N* = 8) was injected ip with the autophagy inhibitor 3-methyladenine (3-MA) at a dose of 100 mg/kg/day for the last 7 days of ethanol or dextrose feeding, the third group (*N* = 8) was injected ip with the autophagy activator rapamycin (Rap) at a dose of 5 mg/kg, once a day for the last 7 days of ethanol or dextrose feeding. At the end of treatment, the mice were terminated, blood collected and serum prepared and liver was removed and cut into aliquots. Some aliquots were used for pathology and H&E staining while other aliquots were stored at −80 °C for preparation of homogenates and further study.

### 4.2. Liver Injury Evaluation

Liver injury after chronic alcohol feeding with or without 3-MA or rapamycin administration was evaluated by determining serum ALT, AST and TG levels; liver H&E staining; and two parameters typically reflective of oxidative stress. ALT and AST levels in the serum was determined with a kit from Pointe Scientific Inc. (Canton, MI, USA) using 15 µL of serum and kinetic determination of absorbance at 340 nm.TG levels were determined with a kit from Pointe Scientific Inc. using 5 µL of serum and assay of absorbance at 540 nm followed by comparison to a standard curve. Liver samples in 10% buffered formaldehyde were processed in a blind fashion by the Mount Sinai Medical Center Pathology Core for H&E staining and pathologic evaluation. Oxidative stress was evaluated by measuring lipid peroxidation as formation of thiobarbituric acid reactive substances (TBARS) and levels of GSH in liver homogenates as described previously [38,41,42].

### 4.3. CYP2E1 Activity and Protein Levels

To evaluate the possible effect of 3-MA and rapamycin directly on CYP2E1, CYP2E1 catalytic activity and protein levels were determined in microsomes isolated from the alcohol and dextrose fed mice of the 3 genotypes. CYP2E1 activity was determined by using *p*-nitrophenol (PNP) as substrate and CYP2E1 protein was determined by immunoblots using a monospecific polyclonal CYP2E1 antibody (a generous gift from Dr. Jerry Lasker ) as described previously [38,43,54].

### 4.4. Levels of Autophagic Proteins

The effect of chronic ethanol or chronic ethanol plus 3-MA or chronic ethanol plus rapamycin administration on levels of Beclin-1, Atg 7 and p62 were evaluated by immunoblots. Antibodies were from Santa Cruz. The immunoblots were scanned and analyzed as the p62/β-actin, Beclin-1/β-actin and Atg 7/β-actin ratios using a LI-COR Odyssey densitometer and software Image J from NIH.

### 4.5. Statistical Analysis

Statistical analysis was performed using one-way analysis of variance with subsequent *post hoc* comparison by Scheffe. Values reflect means ± standard error. The number of experiments is indicated in the figure legends.

## 5. Conclusions

In conclusion, autophagy is protective against ethanol/CYP2E1-dependent toxicity *in vitro* and against acute ethanol/CYP2E1- and chronic ethanol/CYP2E1-dependent toxicity *in vivo*. Since CYP2E1 plays an important role in the toxicity of ethanol, drugs and carcinogens and is activated under various pathophysiological conditions such as diabetes, NASH and obesity, attempts to stimulate autophagy may be beneficial in preventing/lowering CYP2E1/ethanol liver injury.

## References

[1] Klionsky D.J., Emr S.D. (2000). Autophagy as a regulated pathway of cellular degradation. Science.

[2] Shintanl T., Klionsky D.J. (2004). Autophagy in health and disease: A double-edged sword. Science.

[3] Cuervo A.M., Wong E. (2014). Chaperone-mediated autophagy: Roles in disease and aging. Cell Res..

[4] Ding W.X., Manley S., Ni H.N. (2011). The emerging role of autophagy in alcoholic liver disease. Exp. Biol. Med..

[5] Yin X.M., Ding W.X., Gao W. (2008). Autophagy in the liver. Hepatology.

[6] Dong H., Czaja M.J. (2011). Regulation of lipid droplets by autophagy. Trends Endocrinol. Metab..

[7] Czaja M.J. (2010). Autophagy in health and disease. 2. Regulation of lipid metabolism and storage by autophagy: Pathophysiological implication. Am. J. Physiol. Cell Physiol..

[8] Czaja M.J. (2011). Functions of autophagy in hepatic and pancreatic physiology and disease. Gastroenterology.

[9] Singh R., Kaushik S., Wang Y., Xiang Y., Novak I., Komatsu M., Tanaka K., Cuervo A.M., Czaja M.J. (2009). Autophagy regulates lipid metabolism. Nature.

[10] Wu J.J., Quijano C., Wang J., Finkel T. (2009). Metabolism meets autophagy. Cell Cycle.

[11] Rautou P.E., Mansouri A., Lebrec D., Durand F., Valla D., Moreau R. (2010). Autophagy in liver diseases. J. Hepatol..

[12] French S.W., Morimoto M., Reitz R.C., Koop D., Klopfenstein B., Estes K., Clot P., Ingelman-Sundberg M., Albano E. (1997). Lipid peroxidation, CYP2E1 and arachidonic acid metabolism in alcoholic liver disease in rats. J. Nutr..

[13] Morimoto M., Zern M.A., Hagbjork A.L., Ingelman-Sundberg M., French S.W. (1994). Fish oil, alcohol and liver pathology: Role of cytochrome P450 2E1. Proc. Soc. Exp. Biol. Med..

[14] Nanji A.A., Zhao S., Sadrzadeh S.M.H., Dannenberg A.J., Tahan S.R., Waxman D.J. (1994). Markedly enhanced cytochrome P4502E1 induction and lipid peroxidation is associated with severe liver injury in fish oil-ethanol-fed rats. Alcohol. Clin. Exp. Res..

[15] Morimoto M., Hagbjork A.L., Wan Y.J., Fu P.C., Clot P., Albano E., Ingelman-Sundberg M., French S.W. (1995). Modulation of experimental alcohol-induced liver disease by cytochrome P450 2E1 inhibitors. Hepatology.

[16] Gouillon Z., Lucas D., Li J., Hagbjork A.L., French B.A., Fu P., Fang C., Ingelman-Sundberg M., Donohue T.M., French S.W. (2000). Inhibition of ethanol-induced liver disease in the intragastric feeding rat model by chlormethiazole. Proc. Soc. Exp. Biol. Med..

[17] Lieber C.S. (1997). Cytochrome p4502E1: Its physiological and pathological role. Physiol. Rev..

[18] Lu Y., Cederbaum A.I. (2008). CYP2E1 and oxidative liver injury by alcohol. Free Radic. Biol. Med..

[19] Cederbaum A.I., Lu Y., Wu D. (2009). Role of oxidative stress in alcohol- induced liver injury. Arch. Toxicol..

[20] Caro A.A., Cederbaum A.I. (2004). Oxidative Stress, Toxicology and Pharmacology of Cyp2E1. Annu. Rev. Pharmacol. Toxicol..

[21] Raucy J.L., Kraner J.C., Lasker J.M. (1993). Bioactivation of halogenated hydrocarbons by cytochrome P4502E1. Crit. Rev. Toxicol..

[22] Bolt M., Koos P.H., Their R. (2003). The cytochrome P450 isoenzyme CYP2E1 in the biological processing of industrial chemicals. Int. Arch. Occup. Environ. Health.

[23] Koop D.R. (1992). Oxidative and reductive metabolism by cytochrome P4502E1. FASEB J..

[24] Song B.J., Cederbaum A.I., Koop D.R., Ingelman-Sundberg M., Nanji A. (1996). Ethanol-inducible cytochrome P450 (CYP2E1): Biochemistry, molecular biology and clinical relevance Alcoholism. Clin. Exp. Res..

[25] Tanaka E., Terada M., Misawa S. (2000). Cytochrome P450 2E1: Its clinical and toxicological role. J. Clin. Pharm. Ther..

[26] Lieber C.S. (1999). Microsomal ethanol-oxidizing system (MEOS): The first 30 years (1968–1998)—A review. Alcohol. Clin. Exp. Res..

[27] Donohue T.M. (2009). Autophagy and ethanol-induced liver injury. World J. Gastroenterol..

[28] Ding W.X., Li M., Chen X., Ni H.N., Lin C.W., Gao W., Lu B., Stolz D.B., Clemens D.L., Yin X.M. (2010). Autophagy reduces acute ethanol-induced hepatotoxicity and steatosis in mice. Gastroenterology.

[29] Osna N.A., Thomes P.G., Donohue T.M. (2011). Involvement of autophagy in alcoholic liver injury and hepatitis c pathogenesis. World J. Gastroenterol..

[30] Von Haefen C, Sifringer M., Menk M., Spies C.D. (2011). Ethanol enhances susceptibility to apoptotic cell death via down regulation of autophagy-related proteins. Alcohol. Clin. Exp. Res..

[31] Lin C.W., Zhang H., Li M., Xiong X., Chen X., Chen X., Dong X.C., Yin X.M. (2013). Pharmacological promotion of autophagy alleviates steatosis and injury in alcoholic and nonalcoholic fatty liver conditions in mice. J. Hepatol..

[32] Noh B., Lee J.L., Jun H., Lee H.J., Jia Y., Hoang M., Kim J., Park K., Lee S. (2011). Restoration of autophagy by puerarin in ethanol—Treated hepatocytes via the activation of AMP-activated protein kinase. Biochem. Biophys. Res. Commun..

[33] Dolganiuc A., Thomes P.G., Ding W.X., Lemasters J.J., Donohue T.M. (2012). Autophagy in alcohol-induced liver diseases. Alcohol. Clin. Exp. Res..

[34] Eid N., Ito Y., Maemura K., Otsuki Y. (2013). Elevated autophagic sequestration of mitochondria and lipid droplets in steatotic hepatocytes of chronic ethanol-treated rats: An immunohistochemical and electron microscopic study. J. Mol. Histol..

[35] Thomes P.C., Trambly C.S., Thiele G.M., Duryee M.J., Fox H.S., Haorah J., Donohue T.M. (2012). Proteosome activity and autophagosome content in liver are reciprocally regulated by ethanol treatment. Biochem. Biophys. Res. Commun..

[36] Wu D., Wang X., Zhou R., Cederbaum A.I. (2010). CYP2E1 enhances ethanol-induced lipid accumulation but impairs autophagy in HepG2 E47 cells. Biochem. Biophys. Res. Commun..

[37] Wu D., Cederbaum A.I. (2013). Inhibition of autophagy promotes CYP2E1-dependent toxicity via elevated oxidative stress, mitochondrial dysfunction and activation of p38 and JNK MAPK. Redox Biol..

[38] Wu D., Wang X., Zhou R., Yang L., Cederbaum A.I. (2012). Alcohol steatosis and cytotoxicity: The role of cytochrome P4502E1 and autophagy. Free Radic. Biol. Med..

[39] Lu Y., Zhuge J., Wang X., Bai J., Cederbaum A.I. (2008). Cytochrome P4502E1 contributes to ethanol-induced fatty liver in mice. Hepatology.

[40] Ni H.M., Williams J.A., Yang H., Shi Y.H., Fan J., Ding W.X. (2012). Targeting autophagy for the treatment of liver diseases. Pharmacol. Res..

[41] Ni H.M., Du K.D., You M., Ding W.X. (2013). Critical role of FoxO3a in alcohol-induced autophagy and hepatotoxicity. Am. J. Pathol..

[42] Ni H.M., Bockus A., Boggess N., Jaeschke H., Ding W.X. (2012). Autophagy protects against acetaminophen-induced hepatotoxicity. Hepatology.

[43] Yang L., Wu D., Wang X., Cederbaum A.I. (2013). Cytochrome P4502E1, oxidative stress, JNK and autophagy in acute alcohol-induced fatty liver. Free Radic. Biol. Med..

[44] Song B.J., Veech R.L., Park S.S., Gelboin H.V., Gonzalez F.J. (1989). Induction of rat hepatic *N*-nitrosodimethylamine demethylase by acetone is due to protein stabilization. J. Biol. Chem..

[45] Roberts B.J., Shoaf S.E., Song B.J. (1995). Rapid changes in cytochrome P4502E1 activity and other P450 enzymes following ethanol withdrawal in rat. Biochem. Pharmacol..

[46] Song B.J., Gelboin H.V., Park S.S., Yang C.S., Gonzalez F.J. (1986). Complementary DNA and protein sequences of ethanol-inducible rat and human cytochrome P450s: Transcriptional and posttranscriptional regulation of the rat enzyme. J. Biol. Chem..

[47] Huang J., Lam G.Y., Brumell J.H. (2011). Autophagy signaling through reactive oxygen species. Antioxid. Redox Signal..

[48] Shouvel-Scherz R., Elazar Z. (2011). Regulation of autophagy by ROS: Physiology and pathology. Trends Biochem. Sci..

[49] Shouvel-Scherz R., Elazar Z. (2007). ROS, mitochondria and the regulation of autophagy. Trends Cell Biol..

[50] Zalckvar E., Yosef N., Ber Y., Rubinstein A.D., Mor I., Sharan R., Ruppin E., Kimchi A. (2010). A systems level strategy for analyzing the cell death network; implication in exploring the apoptosis/autophagy connection. Cell Death Differ..

[51] Wang Y., Singh R., Xiang Y., Czaja M.J. (2010). Macroautophagy and chaperone-mediated autophagy are required for hepatocyte resistance to oxidant stress. Hepatology.

[52] Liu Z., Lenardo M.J. (2007). Reactive oxygen species regulate autophagy through redox-sensitive proteases. Dev. Cell.

[53] Li L., Ishdorj G., Gibson S.B. (2012). Reactive oxygen species regulation of autophagy in cancer: Implications for cancer treatment. Free Radic. Biol. Med..

[54] Lu Y., Wu D., Wang X., Ward S.C., Cederbaum A.I. (2010). Chronic alcohol-induced liver injury and oxidant stress are decreased in cytochrome P4502E1 knockout mice and restored in humanized cytochrome P4502E1 knock-in mice. Free Radic. Biol. Med..

[55] Gonzalez F.J. (2005). Role of cytochromes P450 in chemical toxicity and oxidative stress: Studies with CYP2E1. Mutat. Res..

[56] Nepal S., Park P.H. (2013). Activation of autophagy by globular adiponectin attenuates ethanol-inducible apoptosis in HepG2 cells: Involvement of AMPK/FoxO3A axis. Biochim. Biophys. Acta.

[57] Lee S.S., Buter J.T., Pineau T., Fernandez-Salguero P., Gonzalez F.J. (1996). Role of CYP2E1 in the hepatotoxicity of acetaminophen. J. Biol. Chem..

[58] Cheung C., Yu A.M., Ward J.M., Krausz J.W., Akiyama T.E., Feigenbaum L., Gonzalez F.J. (2005). The CYP2E1-humanized transgenic mouse: Role of CYP2E1 in acetaminophen hepatoxicity. Drug Metab. Dispos..

